# Sensitization of Gram-negative bacteria to rifampin and OAK combinations

**DOI:** 10.1038/srep09216

**Published:** 2015-03-18

**Authors:** Joanna Jammal, Fadia Zaknoon, Galoz Kaneti, Keren Goldberg, Amram Mor

**Affiliations:** 1 Department of Biotechnology & Food Engineering, Technion-Israel Institute of Technology, Haifa 32000, Israel

## Abstract

While individually inefficient against Gram-negative bacteria, *in-vitro* combinations of rifampin and OAK were mutually synergistic since sub-minimal inhibitory concentrations of one compound have potentiated the other by 2–4 orders of magnitude. Synergy persisted *in-vivo* as single-dose systemic treatment of *Klebsiella* infected mice resulted in 10–20% *versus* 60% survival, respectively accomplished by individual and combined compounds. This outcome was achieved without drug formulation, rather, pharmacokinetic considerations have inspired the therapeutic regimen.

The spread of multidrug resistance (MDR) among pathogenic bacteria continues to challenge modern medicine. In particular, shortage in new antibiotics for treating Gram-negative bacteria (GNB) infections is disquieting, stressing a growing urgency for alternative solutions^1^,^2^,^3^,^4^. Oligo-acyl-lysyls (OAKs) represent a potentially useful approach for developing safe, efficient and economically viable antimicrobial small molecules to meet the global and ever increasing MDR-associated threats^5^,^6^,^7^. Previously, the prototypical OAK sequence acyl-lysyl-lysyl-aminoacyl-lysyl proved to generate OAK derivatives targeting Gram-positive bacteria (GPB)^8^,^9^ and more recently, prompted inefficient antibiotics to improve activity against GNB^10^. Superficial OAK interactions with both the cytoplasmic and outer membrane (CM and OM, respectively, Fig. 1a) were implicated in this chemo-sensitization property, causing naturally resistant bacteria to become sensitive to formerly inactive antibiotics^10^ or to overcome acquired resistance mechanisms^11^.

Here, we challenged these putative OAK-membrane interactions by testing their capacity to mediate uptake of otherwise excluded antibiotics, such as rifampin^12^. Various similar studies were conducted using a panoply of chemicals entities^13^,^14^,^15^,^16^ ranging from cation-chelators to host defense peptides, highlighting the potential usefulness of these combinations that may provide future therapeutic alternatives to GNB infections, albeit not always without dispute^17^. Rifampin and penicillin are hydrophobic antibiotics, respectively targeting cytoplasmic and periplasmic bacterial components. As inactivity of penicillin on GNB is often due to β-lactam processing enzymes, its periplasmic accumulation is not expected to benefit from OAK's action, unlike rifampin whose inefficacy over GNB usually results from its natural low OM-permeability^12^,^18^, although other mechanisms were reported to confer additional resistance to rifampin, including mutations in RNA polymerase gene, *rpoB*^19^,^20^. Note that rifampin (member of the rifamycin family) is a highly effective anti-mycobacterial drug, even though the mycobacterial outer membrane hinders, to some extent, its entry. Only in several mycobacteria species this reduced permeability results in variable degrees of resistance^21^,^22^.

## Results

**Figure 1:**
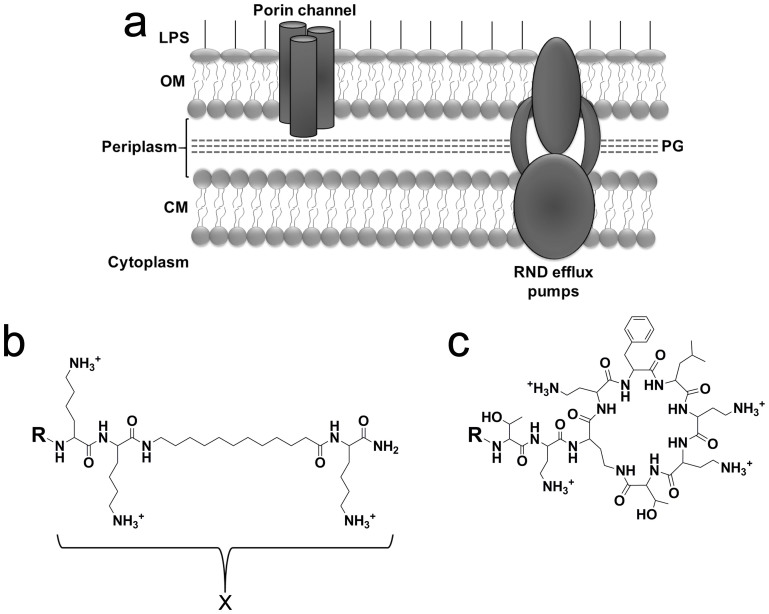
Structural features of bacterial cell wall and lipopeptides. (a) Typical double-membrane organization in Gram-negative bacteria. The outer membrane (OM) is composed of proteins bathing in lipids such as lipoplysaccharides (LPS) in the outermost layer and phospholipids in the inner layer whereas the cytoplasmic membrane (CM) is composed of phospholipids and proteins in both layers. The space between OM and CM (the periplasm) contains a thin peptidoglycan (PG) layer. The protein complex passing through both membranes represents a resistance-nodulation-division efflux pump. (b) Molecular structures of OAKs investigated, referred to as RX, where R is either H or one of the following acyls: octanoyl (C_8_), decanoyl (C_10_), dodecanoyl (C_12_) or its unsaturated version dodecenoyl (C_12(ω7)_) and X is the amino acid sequence lysyl-lysyl-aminododecanoyl-lysyl-amide (KKc_12_K). (c) Structure of polymyxin where R is 6-methyloctanoyl-diaminobutiroyl or H, for the native polymyxin B and nonapeptide derivative, respectively.

**Figure 2:**
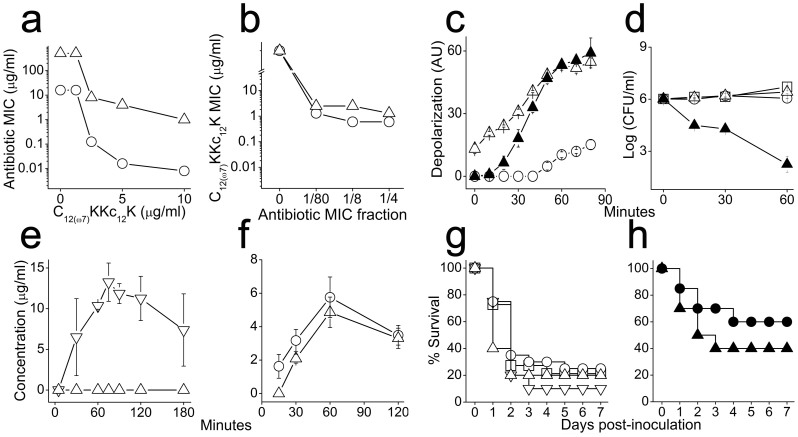
Evidence for sensitization of *E. coli* to antibiotics and OAKs combinations. Panels (a,b) respectively show the MIC evolution when bacteria were treated with rifampin (circles) or erythromycin (triangles) in presence of the specified sub-MIC OAK and *vice versa*. OAK MIC against this strain is >50 μg/ml (panel a). For Panel b, the standalone antibiotic MIC is 16 and 512 μg/ml, respectively for rifampin and erythromycin. Panels (c,d) show the time-dependence of membrane depolarization and the bacterial viability during the experiment, respectively. Symbols: squares, untreated control; circles, rifampin (4 μg/ml); open triangles, OAK (1.3 μg/ml); solid triangles, OAK + rifampin. Panels (e,f) show a pharmacokinetic study using LC/MS analysis to monitor the mean plasma concentrations of C_12(ω7)_X (triangles) and rifampin (inverted triangles) after oral administration of 20 mg/Kg each, calculated based on their respective calibration curves (e) and the same analysis for subcutaneous administrations of C_12(ω7)_X (triangles) and C_10_X (circles) at 12.5 mg/Kg each (f). Error bars = s.d. Panels (g,h) show systemic efficacy in neutropenic mice after monotherapy (g) and combination therapy (h). Symbols: open squares, vehicle treated control; triangles, C_12(ω7)_X alone; circles, C_10_X alone; inverted triangles, rifampin alone; solid triangles, combined treatment of C_12(ω7)_X and rifampin; solid circles, combined treatment of C_10_X and rifampin. The data represent averages from two independent experiments performed with 10 mice/group (standard deviations were <10%).

Minimal inhibitory concentration (MIC) assays demonstrated that OAKs and antibiotics were individually unable to efficiently inhibit growth of GNB, whereas rifampin became extremely potent in presence of sub-MIC of the reference OAK C_12(ω7)_X (Structure shown in Fig. 1b). Figure 2a illustrates the case of *E. coli* while Supplementary Fig. S1 (Supplementary Results online) summarizes data obtained with three additional species representing medically relevant GNB, collectively revealing the extent to which rifampin's MIC was reduced in presence of sub-MIC OAK. Similar data obtained with erythromycin^10^ is included for comparison.

Remarkably, sub-MIC levels of rifampin or erythromycin have also potentiated the OAK's antibacterial activity against *E. coli*, reducing the MIC from >50 down to <1 μg/ml (Fig. 2b). Supplementary Fig. S2 shows that similar outcome is obtained with additional bacterial strains and species, thereby confirming the occurrence of a mutually synergistic process in GNB. Figure 2c,d suggests that sub-MIC OAK and rifampin might induce membrane depolarization at micromolar concentrations, while underlining significant differences in terms of dose and kinetics (mechanistic relevance is discussed below).

Table 1 summarizes the biophysical attributes of OAK derivatives, including published data concerning two derivatives whose N-terminal dodecenoyl was replaced with a saturated version (C_12_X) or deleted^6^,^9^,^23^, indicating that the hydrophobic analog undergoes self-assembly at lower concentrations and is more efficient in hemolysis and antibacterial activities. As evident from the middle part of Table 1, this analog was as potent as C_12(ω7)_X in sensitizing *E.coli* to rifampin. These properties were diminished in absence of the N-terminal acyl, as expected for excessively hydrophilic OAKs^5^,^6^, raising the question of how the OAK would behave if the N-terminal acyl was replaced with analogs having intermediate hydrophobicity values. We therefore produced two new derivatives, C_10_X and C_8_X, which revealed rather intriguing biological profiles: they were less active in hemolytic and antibacterial tests but they sensitized bacteria to rifampin, nonetheless. As shown in Supplementary Table S1, sensitization persisted against different species, thereby establishing C_10_X as a potent sensitizer of GNB to rifampin since it reduced active concentrations from high micromolar- to low nanomolar-range. Note that C_10_X also displayed reciprocal synergism with rifampin (Supplementary Fig. S3) as observed for the reference OAK, whereas these OAKs were unable to sensitize the tested bacteria to penicillin.

Figure 2e,f shows preliminary toxicity and pharmacokinetic data. Oral administrations of OAKs were well tolerated, the maximal tolerated dose (MTD) being >20 mg/Kg, but this dose-range did not allow detectable amounts to reach the blood compartment, unlike rifampin that rapidly accumulated up to 13.26 ± 2.25 μg/ml (nearly as reported)^24^,^25^. In contrast, subcutaneous OAKs administrations (MTD > 20 mg/Kg) were both well tolerated and enabled plasma levels of around 5 μg/ml when dosed at 12.5 mg/Kg. Consequently, to promote optimal/simultaneous blood concentrations during efficacy tests, we opted for subcutaneous OAKs administration- one hour after rifampin's oral administration. To test the drugs ability to affect disease course systemically, we used neutropenic mice to nullify neutrophil-mediated immune contributions in resolving bacterial infections^26^. Inoculation with *K. pneumoniae* followed by vehicle treatment, resulted in rapid death of most mice (20 ± 10% survival) within 1–2 days (Fig. 2g). Under these conditions, single dose treatments with rifampin, C_12(ω7)_X or C_10_X, were unable to significantly improve the survival rates, as they yielded 10, 20 and 25% survivors at day 7 (*P* < 0.004, <0.07 and <0.002, respectively). Administration of rifampin combined with C_12(ω7)_X increased mice survival from 20 to 40% (*P* < 0.003, Fig. 2h), while replacing the reference OAK with C_10_X has further increased the survival rate to 60% (*P* < 0.0004, Fig. 2h).

**Figure 3:**
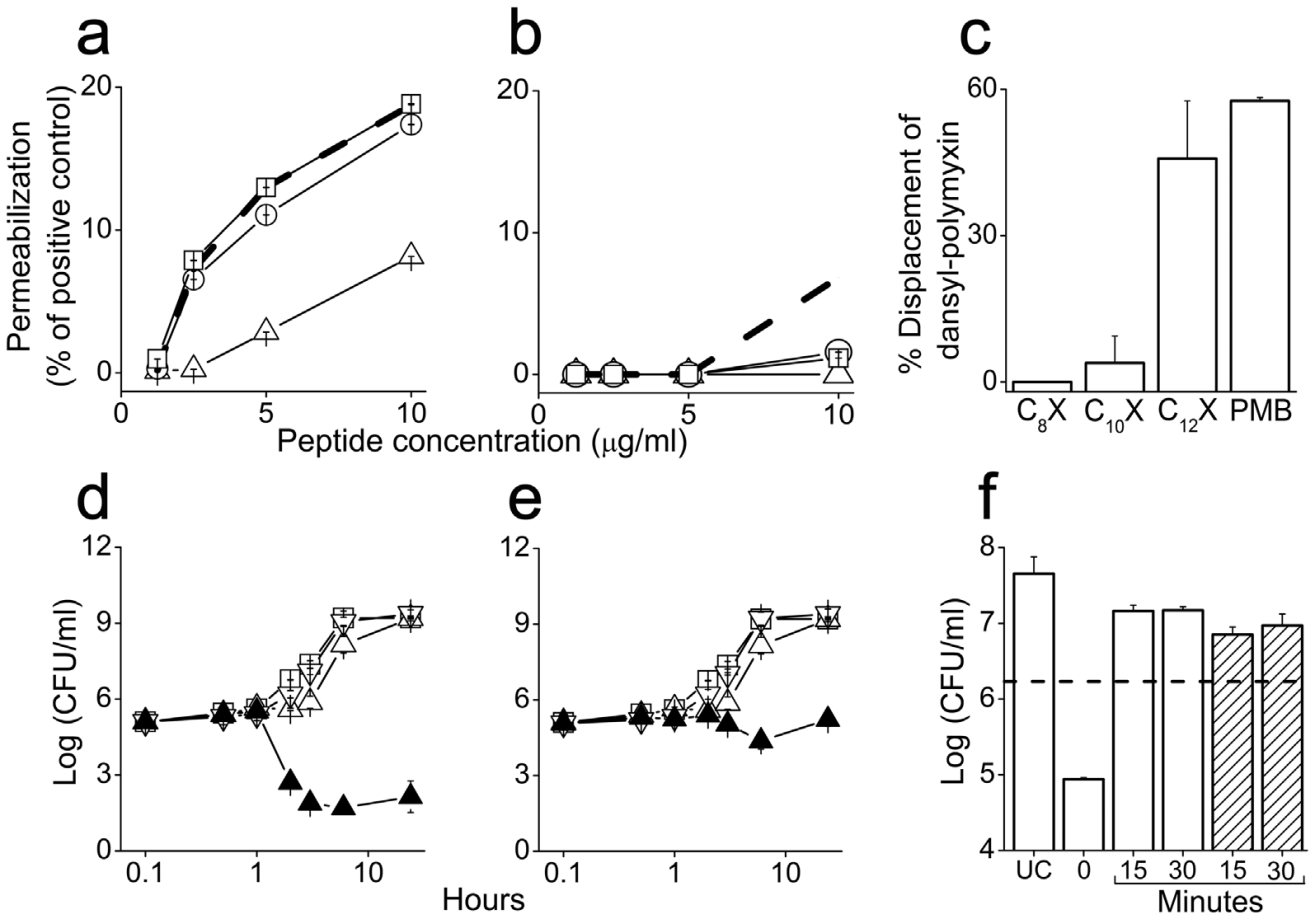
Mechanistic studies. (a,b) Dose-dependent permeabilization of outer and cytoplasmic membranes, respectively, using the *E. coli* mutant ML-35p. Symbols: triangles, C_12(ω7)_X; circles, C_10_X; dashed lines, C_12_X; squares, polymyxin B. Dermaseptin K_4_K_20_-S4 (57 μg/ml) was used for full permeabilization of both membranes. (c) Dansyl-polymyxin binding assay performed after pre-incubation (1.5 h at 10 μg/ml) of OAKs and polymyxin B with 2 μM pure monodansyl-polymyxin and 3 μg/ml LPS from *E. coli*. (d,e) Time-kill kinetics upon bacterial exposure to C_12(ω7)_X alone and in combinations with rifampin (d) and erythromycin (e). Symbols: open squares, untreated control; open triangles, C_12(ω7)_X (5 μg/ml); inverted open triangles, rifampin or erythromycin (0.004 or 0.03 μg/ml, respectively); solid triangles, C_12(ω7)_X + antibiotic. (f) Effect of delayed exposure to rifampin or OAK; Bacteria (*K. pneumoniae* CI 1287) were exposed to both C_12(ω7)_X (5 μg/ml) and rifampin (0.008 μg/ml) without delay (t = 0) and after delayed exposure to rifampin (white bars) or OAK (striped bars) by 15 or 30 min in LB culture medium. CFU counts were determined after additional 3 h incubation in the culture medium. UC; untreated control. Dashed line indicates the inoculum. Error bars = s.d.

To shed light into the molecular basis for the observed synergy, we first utilized an assay capable of differentiating permeability changes in CM and OM by testing the leakage of small molecules in the engineered *E. coli* strain, ML-35p^27^ (a kind gift from Prof. Richard Epand, McMaster University, Canada). The OM became permeable at various sub-MIC values (Fig. 3a) but C_10_X exhibited higher potency than C_12(ω7)_X and was equipotent with polymyxin B, considered gold standard reference for OM permeabilization^13^,^28^,^29^,^30^. At the concentrations used (which were higher than the MIC for polymyxin), the compounds did not premeabilize the CM, except for C_12_X that incidentally, was also more potent than its unsaturated analog (Fig. 3b).

To verify whether the peptides OM permeabilization ability involved similar binding affinities to lipopolysaccharides (LPS), we used the dansyl-polymyxin assay^31^. Supplementary Fig. S4 summarizes the dose-dependent kinetics obtained with LPS from two GNB (*E. coli* and *P. aeruginosa*). Figure 3c shows that OAKs affinity increased with increasing hydrophobicity (Table 1) but the OAKs exhibited a significantly lower affinity than polymyxin B. For instance, about 10-fold difference was observed with C_10_X (P < 0.004). Finally, time-kill kinetics obtained at synergistic concentrations (Fig. 3d,e) with OAK alone and in presence of the biocidal rifampin or the biostatic erythromycin, indicated that after a brief delay, normal bacterial growth has resumed upon exposure to sub-MIC C_12(ω7)_X. This delay was not observable with C_10_X (Supplementary Fig. S5), indicating that this more hydrophilic analog, just like erythromycin or rifampin, did not affect the normal growth rates, only their combination with an OAK did. Moreover, synergism vanished if rifampin and OAKs were not added simultaneously: For example, bacterial survival was nearly normal if rifampin addition was delayed by 15 min after the OAKs (Fig. 3f) and *vice versa* (C_10_X is shown in Supplementary Fig. S5).

## Discussion

Rifampin is often used in combination therapy for treating *Mycobacterium* infections, including tuberculosis and leprosy^32^,^33^. Rifampin is also active on Gram-positive cocci but not on enterobacteriaceae- a large family of GNB that includes common pathogens, such as *E. coli, Klebsiella,* *Salmonella* or *Pseudomonas*. Clearly, the opportunity to expand rifampin's activity spectrum and/or to reduce its adverse effects (e.g., hepatotoxicity), would be welcomed^34^,^35^. The current work revealed that OAKs have the ability to reduce rifampin's active concentrations by several orders of magnitude. Moreover, this study represents the first report (to our knowledge) of a reciprocal chemo-sensitization (mutual synergism) process of such a magnitude, imparting potency upon two molecular species acting by distinct mechanisms, hence the efforts invested towards understanding the underlying molecular basis.

We first focused on obtaining evidence susceptible to clarify the individual roles in the synergistic pair. The activities exhibited by C_12_X alone, might partly explain the enhanced potency observed upon combination. However, being virtually devoid of antibacterial activity on its own, chemo-sensitization activity associated with C_10_X is a *priori* unexpected; thus, synergism is achievable with little regard to individual antibacterial capacity. In that sense, the new analogs represent valuable tools for deciphering the mechanism underlying synergy by assigning individual responsibilities of each reactant. The fact that C_10_X was more potent than C_12(ω7)_X in permeabilizing the OM is consistent with the view that hemolysis and antibacterial activities are distinguishable from chemo-sensitization. Hence, by reducing their hydrophobicity, C_10_X and C_8_X may have lost attributes that mediated one type of activity but not the other. Consequently, the data can be interpreted as suggesting that, in principle, each synergistic partner does “his own thing”, i.e., C_10_X does not participate in the antibiotic effect (which is the sole responsibility of rifampin) but merely enables rifampin to overcome the obstacle preventing its interaction with RNA polymerase, the OM barrier. The biocidal effect observed upon combining OAK and rifampin (as opposed to biostatic effect with OAK and erythromycin), supports this view. Also supporting this hypothesis is the finding that optimal sensitization of rifampin was reached at ng/ml as opposed to μg/ml for OAKs. These concentration ranges are consistent with the drugs specific and non-specific modes of action, respectively.

The mechanism enabling antibiotics to potentiate the OAKs is less understood. It seems to result from precursor damages exerted by micromolar sub-MIC antibiotics that somehow produce OAK-hypersusceptible bacteria. This may occur with rifampin by competing with OAKs for membrane interactions due to its hydrophobic/cationic characters, as suggested by the sigmoidal shape of the depolarization curve which was observed only at micromolar combinations of OAK-rifampin. The sigmoidal shape disappeared at nanomolar levels of rifampin (data not shown) or upon replacing rifampin with micromolar levels of the porin-gated erythromycin^10^. Notwithstandingly, reciprocal synergism might also occur between erythromycin and OAKs, should these efflux substrates^10^ hamper each other's extrusion by populating the binding pockets of resistance-nodulation-division (RND) pumps^36^. Future studies might clarify this issue.

Of interest is the comparison between OAKs and polymyxins, since these cationic lipopeptides permeabilize the OM with similar potencies (Fig. 3a) despite major differences in chemo-physical attributes (Fig. 1c). OAK derivatives reduced rifampin's MIC by 4000 folds at 5 μg/ml (8000-fold at 10 μg/ml, data not shown). Under similar conditions, polymixin B nonapeptide derivatives have reportedly reduced rifampin's MIC by 85–750 and 250–500 folds, respectively against *E. coli* and *K. pneumoniae* strains^37^,^38^. The large difference in sensitization factors suggests that it is not solely due to OM permeabilization, perhaps it is also related to additional factors, including the way each lipopeptide affects the CM structure and function^9^,^10^. In this regard, polymyxins maybe rather handicapped because of their higher affinity for LPS.

Also, this study showed that certain OAKs enhance the effect of rifampin as already described for other antimicrobials (e.g., defensins^39^) thus raising the question of eventual OAKs advantages compared to antimicrobials that form pores that may allow a better passage of the antibiotic. The advantages of OAKs and other peptidomimetics^40^,^41^,^42^ over classical antimicrobial peptides and proteins are largely documented in the literature and the structural differences compared to polymyxins are highlighted in Fig. 1b,c and Table 1, including in terms of overall size, chemophysical and structural complexity. Besides these differences, antimicrobials that exert “heavy damages” to bacterial membranes are bactericidal and might not necessarily sensitize to rifampin (or other antibiotics) to a great extent as shown with polymyxin B. There is also an inherent advantage over lytic/bactericidal compounds since the milder action of bacteriostatic compounds such as the OAKs in question, reduces the risk for complications associated with endotoxins release^43^,^44^.

The question of whether synergism can effectively occur in animals was addressed by comparing the drugs efficacies in rescuing critically ill mice. Our pharmacokinetic data suggest that the relevant drug concentrations are achievable at sub-MTD doses whereas the notion that synergism observed *in-vitro* could persist *in-vivo* is supported by the viability outcome (Fig. 2h). Therefore, by intimately linking the pharmacokinetics of small molecules and their reciprocal synergism, this study provides encouraging evidence for the potential medical usefulness of the OAK approach for combating human and animal infectious diseases caused by GNB.

## Methods

### Peptide synthesis

The OAKs were prepared in-house by the solid-phase method^45^, applying the Fmoc (9-fluorenylmethyloxycarbonyl) active ester chemistry using automated peptide synthesizer (model 433A; Applied Biosystems, Foster City, CA, USA) as described^5^.

### Organization in solution

OAKs self-assembly in solution was assessed by light-scattering measurements. OAKs at initial concentration of 100 μM were submitted to serial 2-fold dilutions in phosphate buffered saline (PBS) (10 mM Na_2_HPO_4_, 150 mM NaCl, pH = 7.0) and incubated for 2 h at room temperature (RT) and light scattering of each dilution was recorded by holding both the excitation and the emission at 400 nm (slit width, 1 nm). The data represent averages of at least two separate recordings.

### Bacteria

Gram-negative species tested: *Escherichia coli* (ML-35p; American Type Culture Collection (ATCC; Manassas,VA, USA) strain: β-lactamase producer 35218; clinical isolates: 14182, 14384, U16327, U16329), *Pseudomonas aeruginosa* (CI 11662), *Salmonella enterica* serovar Typhimurium (ATCC 14028), *Salmonella enterica* serovar Choleraesuis (ATCC 7308), *Klebsiella pneumoniae* (CI 1287) and carbapenemase-2 producing strain of *Klebsiella pneumoniae* (KPC-2).

### Antibacterial assays

Minimal inhibitory concentrations (MICs) were determined in sterilized 96-well plates. Bacteria were grown overnight in Tryptic Soy Broth (TSB) or Luria-Bertani (LB) broth and adjusted to 10^6^ CFU/ml. Next, 100 μl growth medium containing bacteria was added to 100 μl culture medium containing the test compound in serial 2-fold dilutions. Proliferation inhibition was determined by optical density measurements (620 nm) after incubation overnight at 37°C.

Chemo-sensitization was assessed similarly, except that bacteria were incubated with a mixture of OAK and antibiotics.

Bactericidal kinetics were determined in a final volume of 1 ml, as follows: 100 μl of suspension containing bacteria at 10^6^ CFU/ml were added to 900 μl of culture medium containing zero or various concentrations of OAK, antibiotic, or their combination. After the specified time-points of exposure (37°C under shaking), cultures were subjected to serial 10-fold dilutions in saline (0.85% NaCl) and plated for bacterial enumeration after additional 24 h incubation. Data were obtained from at least two independent experiments.

Effects of drug delay were assessed similarly, except that bacteria were pre-incubated with OAK for 15 or 30 min, centrifuged and re-suspended in fresh LB containing rifampin and incubated for 3 h before CFU enumeration.

### Membrane depolarization

Measurements were performed with 3,3-dipropylthiadicarbocyanine iodide (DiSC_3_5), a lipophilic potentiometric dye^46^. Cells were treated with 2 mM EDTA prior to addition of DiSC_3_5 stock solution (final concentration 0.4 μM) and quenching at RT for 20–30 min. KCl was then added (final concentration 100 mM), the suspension incubated overnight (4°C), 180 μl aliquots were placed in black 96-well plate for 30 min to allow stabilization of the dye signal, then 20 μl of stock solutions of OAK, rifampin, or their combination, were added to obtain the desired final concentrations. Membrane depolarization was monitored by measuring exaction/emission at 620/680 nm, respectively, under shaking at 37°C (BioTeK Synergy HT Microplate Reader). Data were obtained from at least two independent experiments performed in duplicate.

### Outer and inner membrane permeabilization

The mutant *E. coli* ML-35p was used to monitor the ability of the OAK to perforate/perturb the inner and outer membranes^27^. The assay was performed in sterile 96-well plates in a final volume of 200 μl. Bacteria were grown overnight in TSB, washed 3 times in sodium phosphate buffer (SPB) 10 mM, pH = 7.4 and diluted to 10^7^ CFU/ml in SPB containing 3% TSB. Aliquots of this suspension (100 μl) were added to 100 μl of SPB containing a test compound and either *ortho*-nitrophenyl-β-galactoside (ONPG; 2.5 μM) or nitrocefin (25 μM). Hydrolysis of ONPG and nitrocefin was monitored by measuring absorbance at 420 or 486 nm, respectively, at various time intervals, with shaking at 37°C (BioTeK Synergy HT Microplate Reader). Data were obtained from at least two independent experiments performed in duplicate.

### Dansyl-polymyxin binding assay

The affinity of OAKs to LPS from *E. coli* or from *P. aeruginosa* was studied by displacement of bound dansyl-polymyxin, as described^31^. Briefly, Polymyxin B sulphate was dansylated using dansyl chloride followed by mono dansyl-polymyxin B (DPMB) purification by HPLC. The assay was performed in black 96-well plates containing 180 μl of HEPES (5 mM, pH = 7.2), 3 μg/ml LPS, 2 μM mono-DPMB and 20 μl of OAK or polymyxin solution in the desired concentrations (0.6–10 μM). The mixtures were incubated for 1.5 h (RT). The displacement of DPMB was measured as the corresponding decrease in fluorescence (exaction/emission at 340/485 nm) using a BioTeK Synergy HT Microplate Reader. Data were obtained from at least two independent experiments performed in duplicate.

### Hemolytic assay

Hemolytic activity was assessed using fresh Human blood collected into sodium-citrate-containing test tubes, rinsed 3 times in PBS (centrifuged at 200 × *g* for 2 min). Packed cells were re-suspended in PBS resulting in 1% hematocrit. 50 μl of this suspension were added to Eppendorf test tubes containing 200 μl of test compound solutions (in serial twofold dilutions), PBS alone (for base-line value), or double distilled water (DDW) (for 100% hemolysis). After 3 h incubation (37°C under shaking), samples were centrifuged at 14000 × *g* for 2 min and hemolytic activity was assessed as function of hemoglobin leakage by measuring absorbance of 200 μl of supernatant at 450 nm.

### *In-vivo* studies

Animal studies were performed using male ICR mice (weight range, 23 ± 2 g) obtained from Harlan Laboratories (Rehovot, Israel). Procedures, care and handling of animals were reviewed and approved by Technion Animal Care and Use Committee.

### Toxicity

Maximal tolerated dose (MTD) was determined after single-dose subcutaneous (SC) or oral (gavage) administration of OAKs at specified doses using 2 or 10 mice/compound. Animals were inspected for adverse effects during 6 h by recording motor activity, piloerection, redness in ear lobes, cyanosis, protruding eyeballs, slow or labored breathing, loss of response in the rear leg and convulsions. Mortality was monitored during 7 days thereafter.

### Pharmacokinetic study

Drugs blood concentrations were determined by LC-MS using calibrated curves essentially as described^5^,^9^. Briefly, OAK and/or rifampin were given by SC or oral administration. At specified time intervals mice were euthanized (CO_2_ asphyxiation) and blood samples collected from vena cava (N = 2 mice/time point). For analysis, samples were centrifuged (2 min, 6000 × *g*), 200 μl plasma were mixed with 0.5 ml extraction buffer (50% acetonitrile (ACN): 50% methanol) incubated (30 min on plate shaker at 200 rpm, RT), centrifuged (2 min, 14000 × *g*) and 300 μl supernatant diluted twofold in DDW and analyzed by LC-MS (5 μl injected to Waters Xevo G2 Tof/ACQUITY UPLC H-Class system). Flow rate, 0.5 ml/min. Run time, 5 min. Mobile phase, ACN/DDW combination containing 0.1% formic acid using an ACQUITY UPLC BEH column (C_18_ 1.7 μm) and eluted with a 0–90% ACN gradient. Quantification was by MS detection in positive ionization mode using an identical procedure that was performed in mouse whole blood in order to establish standard calibration curves.

### Efficacy

Mice were rendered neutropenic by intraperitoneal (IP) injection of cyclophosphamide (150 and 100 mg/Kg on days 0 and 3, respectively). The procedure was confirmed to result in severe neutropenia by day 4, at which time infection was induced. The peritonitis-sepsis model was used whereby infection was obtained after IP injection of a logarithmic-phase culture of *K. pneumoniae* (3 × 10^7^ CFU/mice in 0.3 ml PBS). Immediately thereafter, mice were treated orally with rifampin (0.25 ml DDW containing 0.45 mg/mouse), whereas the OAKs were administered subcutaneously, an hour post-inoculation (0.3 ml PBS containing 0.3 mg/mouse). Mice survival was monitored for up to **7** days post-treatment. Survival data were obtained from 2 independent experiments (n = 10 mice/group/experiment). Statistical analysis was performed using a paired *t test* were α equals 0.05.

## Author Contributions

J.J. synthesized reagents, performed research, analyzed data, wrote the paper; F.Z. performed research (*in-vivo* blood concentrations), analyzed data; G.K. performed research (*in-vivo* toxicity and efficacy experiments), analyzed data; K.G. performed research (part of the antibacterial assays); A.M. designed the experiments, analyzed data, wrote the paper. All authors discussed the results and commented on the manuscript.

## Supplementary Material

Supplementary Information Supplementary Information

## Figures and Tables

**Table 1:** Structure-activity study highlighting biophysical attributes of N-terminal OAK derivatives

Sequence	%H^a^	Q^b^	CAC^c^ (μM)	LC_50_^d^ (μM)	MIC^e^ (μM)	MIC of rifampin in presence of OAK (μg/ml)					SF	MIC of penicillin G in presence of OAK (μg/ml)				SF
						0	1.25	2.5	5			0	1.25	2.5	5	
**C_12(ω7)_X**	**49**	**3**	**40**	**100 ± 8**	**≥50**	8–16	8–16	≤0.06		0.004	2000–4000	>512	>512	>512	>512	1
**C_12_X**	**51**	**3**	**12.5**	**29 ± 9**	**16**	8–16	8–16	≤0.06		0.002	4000–8000	>512	>512	>512	>512	1
C_10_X	46	3	≥100	>100	>50	8–16	4	0.02		0.004	2000–4000	>512	>512	>512	>512	1
C_8_X	41	3	≥100	>100	>50	8–16	2	0.5		0.06	133–267	>512	>512	>512	>512	1
**X**	**27**	**4**	**≥100**	**>100**	**>50**	8–16	8–16	8–16		8	1–2	>512	>512	>512	>512	1

X = lysyl-lysyl-aminododecanoyl-lysyl-amide;

^a^Hydrophobicity measure, defined as % acetonitrile eluent in C_18_ HPLC column;

^b^Molecular charge in physiological conditions;

^c^Critical aggregation concentration in PBS;

^d^OAK concentration that induced 50% hemolysis after 3 h incubation in PBS 37°C;

^e^Tested against *E. coli* strain ML-35p. SF, sensitization factor defined as the ratio of the MIC in absence of OAK to that in presence of 5 μg/ml of OAK. Bold lines indicate published data.
