# Supplementary material for: Sensitization of Gram-negative bacteria to rifampin and OAK combinations
Source: Sci Rep. 2015 Mar 18;5:9216. doi: 10.1038/srep09216 (PMC4363860; doi:10.1038/srep09216)
Supplement: Supplementary Information [file srep09216-s1.pdf]

Sensitization of Gram-negative bacteria to rifampin and OAK combinations

Joanna Jammal[1], Fadia Zaknoon[1], Galoz Kaneti[1], Keren Goldberg[1], Amram Mor[1*]

1 Department of Biotechnology & Food Engineering, Technion-Israel Institute of Technology, Haifa 32000, Israel.

* Correspondence: Amram Mor[1] e-mail: amor@tx.technion.ac.il

Supplementary Figure S1: Evidence for sensitization of GNB to antibiotics by $C_{12(\omega7)}KKc_{12}K$. Shown are the MIC evolutions when bacteria were treated with rifampin (circles) or erythromycin (triangles) in presence of the specified sub-MIC OAK. OAK MIC against these strains is >50 µg/ml.

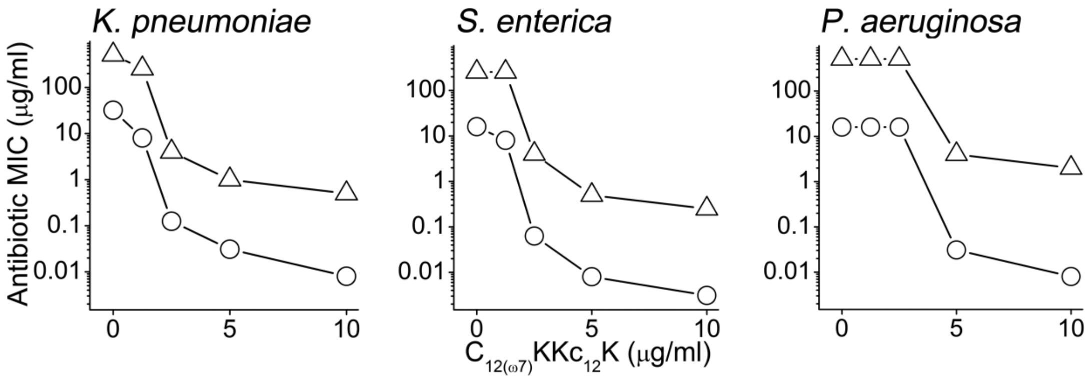

Supplementary Figure S2: Evidence for sensitization of GNB to $C_{12(\omega 7)}KKc_{12}K$ by antibiotics. Shown are the OAK's MIC evolutions when bacteria were treated with the specified sub-MIC of rifampin (circles) or of erythromycin (triangles). The standalone rifampin MIC against *E. coli* and *Klebsiella* is 16 and 32 µg/ml, respectively, and 512 µg/ml for erythromycin.

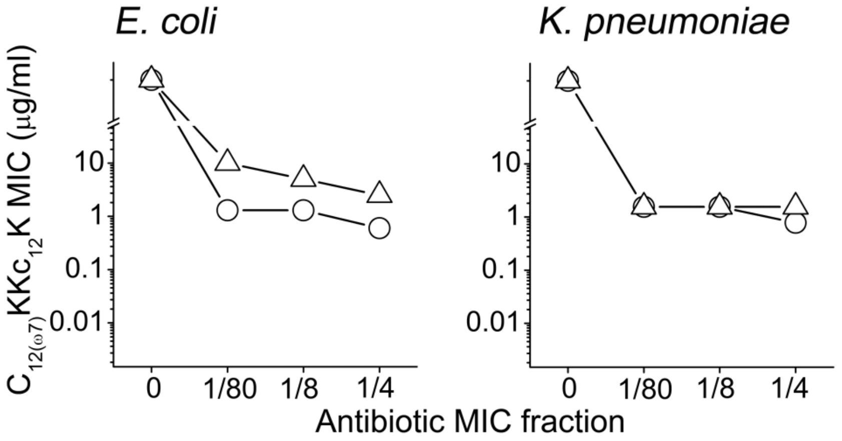

Supplementary Figure S3: Evidence for sensitization of GNB to $C_{10}KKc_{12}K$ by antibiotics. Shown are the OAK's MIC evolutions when bacteria were treated with the specified sub-MIC of rifampin (circles) or of erythromycin (triangles). The standalone rifampin MIC against *E. coli* and *Klebsiella* is 16 and 32 µg/ml, respectively, and 512 µg/ml for erythromycin.

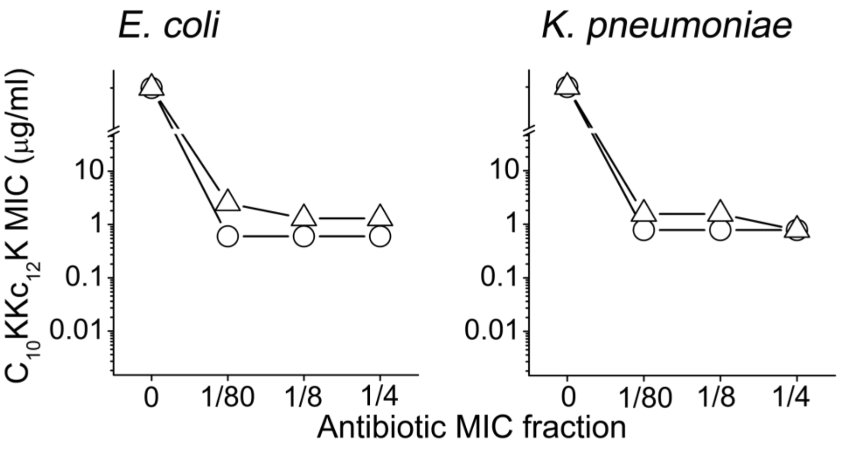

Supplementary Figure S4: Dansyl-polymyxin binding assay. OAKs and polymyxin B were incubated (1.5 h) with 2 µM mono-dansyl-polymyxin and 3 µg/ml LPS from *E. coli* (**a**) or *P. aeruginosa* (**b**). Symbols: squares, polymyxin B; triangles, $C_{12}KKc_{12}K$; circles, $C_{10}KKc_{12}K$; diamonds, $C_{8}KKc_{12}K$. Error bars = s.d.

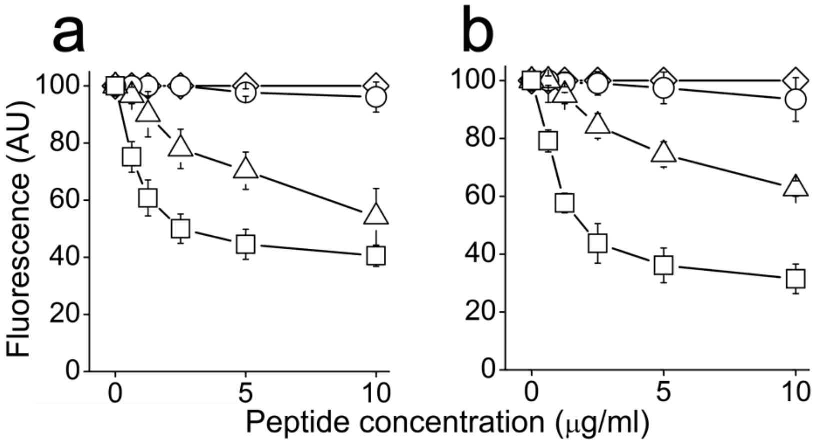

Supplementary Figure S5: Mechanistic studies. Panels (**a,b**) show time-kill kinetics upon bacterial exposure to $C_{10}X$ alone and in combinations respectively with rifampin (**a**) and erythromycin (**b**). Symbols: open squares, untreated control; open circles, $C_{10}X$ (5 µg/ml); inverted open triangles, rifampin or erythromycin (0.004 or 0.03 µg/ml, respectively); solid circles, $C_{10}X$ + antibiotic. Panels (**c**) shows the effect of delayed exposure to rifampin or OAK; Bacteria (*K. pneumoniae* CI 1287) were exposed to both $C_{10}X$ (5 µg/ml) and rifampin (0.008 µg/ml) without delay (t = 0) and after delayed exposure to rifampin (white bars) or OAK (striped bars) by 15 or 30 min in LB culture medium. CFU counts were determined after additional 3 h incubation in the culture medium. UC; untreated control. Dashed line indicates the inoculum. Error bars = s.d.

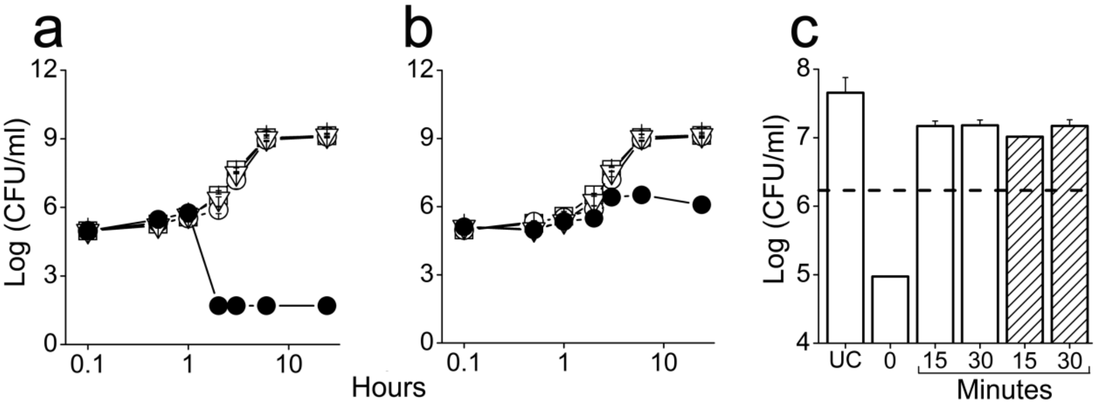

Supplementary Table S1: Antibiotics activities against a panel of GNB in presence of sub-MIC levels of $C_{12(\omega7)}X$ and $C_{10}X$.

Antibiotic	GNB strain	MIC of antibiotic in presence of $C_{12(\omega7)}KKc_{12}K$ (µg/ml)				SF	MIC of antibiotic in presence of $C_{10}KKc_{12}K$ (µg/ml)				SF
		0	1.25	2.5	5		0	1.25	2.5	5	
	E. coli 35218										
	E. coli 14182										
	E. coli 16327										
	E. coli 16329										
Penicillin G	*E. coli* 14384	>512	>512	>512	>512	1	>512	>512	>512	>512	1
	S. Typhimurium 14028										
	S. stanley 7308										
	K. pneumoniae 1287										
	KPC2										
Rifampin	*E. coli* 35218	ND	ND	ND	ND	ND	8	0.5	0.063	0.031	258
	E. coli 14182	8	8	0.031	<0.01	>800	16	4	0.125	0.031	516
	E. coli 16327	16-32	8	<0.01	<0.01	>3200	16	0.5	0.031	0.008	2000
	E. coli 16329	16	8	0.031	0.008	2000	16	0.5	0.063	0.008	2000
	E. coli 14384	ND	ND	ND	ND	ND	8	0.5	0.125	0.031	258
	S. Typhimurium 14028	16	8	0.016	0.006	2667	16	8	0.063	0.008	2000
	S. stanley 7308	ND	ND	ND	ND	ND	16	16	0.125	0.008	2000
	K. pneumoniae 1287	32	4	0.1	0.006	5333	32	8	0.031	0.008	4000
	KPC2	ND	ND	ND	ND	ND	32	32	0.063	0.008	4000

MIC of $C_{12(\omega7)}X$ and $C_{10}X$ against the listed strains was ≥50 µg/ml. SF, sensitization factor. ND, not determined.
